# Heparan sulphate synthetic and editing enzymes in ovarian cancer

**DOI:** 10.1038/sj.bjc.6603747

**Published:** 2007-04-17

**Authors:** A C Backen, C L Cole, S C Lau, A R Clamp, R McVey, J T Gallagher, G C Jayson

**Affiliations:** 1 Department of Medical Oncology, Paterson Institute for Cancer Research, Christie Hospital, Cancer Research UK and University of Manchester, Wilmslow Road, Manchester M20 4BX, UK; 2 Department of Histopathology, St Mary's Hospital, Oxford Road, Manchester M13 0JH, UK

**Keywords:** ovarian cancer, HS, FGF, heparanase, HSULF-1

## Abstract

Several angiogenic growth factors including fibroblast growth factors 1 and 2 (FGF1 and FGF2) depend on heparan sulphate (HS) for biological activity. We previously showed that all cellular elements in ovarian tumour tissue synthesised HS but biologically active HS (i.e. HS capable of binding FGF2 and its receptor) was confined to ovarian tumour endothelium. In this study, we have sought to explain this observation. Heparan sulphate sulphotransferases 1 and 2 (HS6ST1 and HS6ST2) attach sulphate groups to C-6 of glucosamine residues in HS that are critical for FGF2 activation. These enzymes were strongly expressed by tumour cells, but only HS6ST1 was found in endothelial cells. Immunostaining with the 3G10 antibody of tissue sections pretreated with heparinases indicated that HS proteoglycans were produced by tumour and endothelial cells. These results indicated that, in contrast to the endothelium, HS produced by tumour cells may be modified by cell-surface heparanase (HPA1) or endosulphatase (SULF). Protein and RNA analysis revealed that HPA1 was strongly expressed by ovarian tumour cells in eight of ten specimens examined. HSULF-1, which removes specific 6-O-sulphate groups from HS, was abundant in tumour cells but weakly expressed in the endothelium. If this enzyme was responsible for the lack of biologically active HS on the tumour cell surface, we would expect exogenous FGF2 binding to be preserved; we showed previously that this was indeed the case although FGF2 binding was reduced compared to the endothelium and stroma. Thus, the combined effects of heparanase and HSULF could account for the lack of biologically active HS in tumour cells rather than deficiencies in the biosynthetic enzymes.

The extracellular matrix (ECM) is composed of a network of macromolecules that maintain tissue and cellular architecture. Heparan sulphate (HS) proteoglycans (HSPGs) are major components of the ECM and of cell-surface membranes, and they play critical roles in regulating the flow of information between cells and their microenvironment (Blackhall *et al*, 2001). In addition to anchoring cells to matrix fibres and maintaining tissue structure, HSPGs serve at least two additional biological activities. First, the HS chains of these PGs bind to many growth factors and cytokines such as fibroblast growth factors (FGFs), vascular endothelial growth factor and transforming growth factor (Lyon *et al*, 1997; Robinson and Stringer, 2001; Ostrovsky *et al*, 2002, respectively). This binding confers on the growth factors resistance to proteolysis and biological stability and provides a reservoir of growth and migration factors that can be mobilised in accordance with physiological demand. Second, cell-surface HSPGs act as mandatory co-receptors, facilitating binding of growth factors to their cognate high-affinity signal-transducing receptors (reviewed in Gallagher, 2001; Presta *et al*, 2005).

Fibroblast growth factor 2 is a potent angiogenic cytokine, which is dependent on HS for its biological activity (Yayon *et al*, 1991, Chang *et al*, 2000). In a previous study of HSPGs, we showed that stromal syndecan 1 was an adverse prognostic factor for ovarian cancer and that syndecan 3 was unusually expressed by the vasculature (Davies *et al*, 2004). However, the majority of the function of the HSPGs is mediated by the structure of the HS glycosaminoglycan component (Jayson *et al*, 1998). Therefore, in a second study, we investigated the relationship between HS, FGF-2 and the signal-transducing receptors in human, advanced-stage, serous ovarian adenocarcinoma. Using a unique molecular probe, FR1c-AP, which consisted of a soluble FGF receptor 1 isoform IIIc covalently linked to an alkaline phosphatase moiety, the distribution of HS that had the ability to support the formation of an HS/FGF-2/FR1c-AP complex was determined (Ornitz *et al*, 1992; Chang *et al*, 2000). This may be taken as a surrogate marker for the distribution of biologically active HS. In ovarian cancer tissue, we found that this probe bound predominantly to endothelial cells and stroma but not adenocarcinoma cells (Whitworth *et al*, 2005). In the current study, we therefore tested the hypothesis that the enzymes that sulphate HS, such as the sulphotransferases and the HS-cell-surface editing enzymes, such as the endosulphatases and endoheparanase (HPA1), determine the distribution of FGF2-activating HS.

Heparan sulphate chains are synthesised as a post-translational modification of specific core proteins in the Golgi by enzymes that initially polymerise a chain of repeating glucuronic acid (GlcA) and GlcNAc disaccharides, which are then variously modified by N- and O-sulphation and hexuronic acid epimerisation, converting glucuronic acid (GlcA) into iduronic acid (Lindahl *et al*, 1998). These modifications occur in clusters, creating polymorphic sulphated domains (S-domains) that form the principal recognition sites for heparin/HS-binding proteins.

A key modification of HS is carried out by the small family of 6-O-sulphotransferases (HS6ST) that catalyse the transfer of sulphate from adenosine 3′-phosphate, 5′-phosphosulphate to C-6 (exocyclic carbon) of the glucosamine residue in HS, thereby providing functional groups that are critical to the formation of the trimolecular signalling complex: FGF2, FGFR and HS (Pye *et al*, 1998; Robinson *et al*, 2005; Ai *et al*, 2006). The S-domains containing 6-O-sulphate groups can be excised from the HS chain by cell-surface heparanase (HPA1), a *β*-endoglycosidase that releases saccharide fragments from HSPG core proteins. Alternatively, the fine structure of HS can be modified by endosulphatases that specifically remove 6-O-sulphate groups from HS-S-domains (Morimoto-Tomita *et al*, 2002; Viviano *et al*, 2004). This has functional consequences as, in quails, QSULF-1, the avian homologue of mammalian SULFs, has dual regulatory functions as a negative regulator of FGF signalling and a positive regulator of Wnt signalling (Ai *et al*, 2003; Wang *et al*, 2004)

Heparanase mRNA expression has been investigated in a wide variety of human tumours (reviewed in Simizu *et al*, 2004). Kodama *et al* (2003) examined heparanase RNA expression by RT–PCR in five borderline and 31 malignant epithelial ovarian tumours. Heparanase mRNA expression was present in 16 of 31 malignant epithelial ovarian tumours. In contrast, in the five borderline epithelial ovarian tumours, heparanase mRNA was not detected.

In the current study, we have investigated why biologically active HS is largely restricted to the ovarian tumour endothelium. The data suggest that tumour cells synthesise HS, but its binding properties may be modified by SULF action. Moreover, the pattern of expression of heparanase reveals the potential for localised degradation of tumour-associated HS, and release of active HS saccharide-growth factor complexes that could enhance the malignant phenotype.

## MATERIALS AND METHODS

### Tissue samples

Tumours from 10 patients with serous ovarian carcinomas were investigated; these were a mixture of well, moderately and poorly differentiated tumours. Two normal ovaries were also examined. These were taken from consenting patients, under the ethical permission of South Manchester Research Ethics Committee.

### Riboprobe preparation

Specific riboprobes for HS6ST1, HS6ST2, HPA1 and HSULF-1 were prepared by amplifying gene-specific fragments, identified using the NCBI Blast programmes, from normal ovarian RNA using the primers as follows:

HS6ST1 forward 5′-AAAGATATCATGGTTGAGCGCCGC-3′ and reverse 5′-CTCGCGGACCGGGAAGTAG-3′, HS6ST2 forward 5′-GAATTCGGCCAGGCGAAAGCGTC-3′ and reverse 5′-GTCGACCGCCATTTCTCTACACTG-3′, HPA1 forward 5′-TTCGATCCCAAGAAGGAATCAAC-3′ and reverse 5′-GTAGTGATGCCATGTAACTGAATC-3′, HSULF-1 forward 5′-GGATCCCCTTCCACGCTCTGGCCGATTG-3′ and reverse 5′-GGATCCATCCAACAGTCAAATCACTTGCCCAAAT-3′.

These were cloned into the pSPT19 vector (Roche, Mannheim, Germany) and the sequence was verified. The vector was linearised, rendered RNAase-free by phenol/chloroform/isoamyl alcohol purification and used as a template for *in vitro* transcription of digoxigenin-labelled antisense or sense (control) riboprobes using a SP6/T7 transcription kit (Roche).

### ISH method

Tissue sections were dewaxed and rehydrated, denaturated with 0.2 M HCl for 20 min and then digested with proteinase K (5 *μ*g ml^−1^) at 37°C for 30 min. Slides were hybridised with the anti-sense probe or sense probe (negative control) at concentrations of 1–2 *μ*g ml^−1^ in hybridisation buffer (Sigma-Aldrich, Gillingham, UK). After hybridisation, washes and incubation with anti-digoxigenin antibody and visualisation were carried out using NBT/BCIP (Roche) according to the manufacturer's instructions. Sections were fixed, mounted and examined with an Olympus BX51 light microscope (Olympus, Southall, UK) using a × 40 plan neofluar, 1.35 NA, oil immersion objective lens. Visualisation was carried out utilising a Progress C14 camera (Jenoptik, Jena, Germany) via Adobe PhotoShop 7.

### Streptavidin ABC/HRP Immunohistochemistry

Tissue sections were dewaxed and rehydrated. Antigen retrieval was performed in citrate buffer (pH6), using a microwave oven. For 3G10 staining only, sections were pre-treated with heparinases I and II (Grampian Enzymes, Orkney, UK) at a total of 5 mU ml^−1^ in 50 mM sodium acetate, 0.5 mM calcium acetate (pH 7.0), for 4 h at 37°C, replenishing after 2 h. To demonstrate the specificity of 3G10 binding, a negative control section was included in which the heparinases I and II digestion step had been omitted. Endogenous peroxidase activity was blocked by incubation in 0.8% H_2_O_2_ in methanol for 10 min. A blocking solution of 10% goat serum in TBS was applied for 10 min. Sections were incubated overnight at 4°C with either anti-HS monoclonal antibody 10E4 (Seikagaku Corporation, Tokyo, Japan) at a dilution of 1 : 200 (5 *μ*g ml^−1^ final concentration), anti Δ-HS monoclonal antibody 3G10 (Seikagaku Corporation) at a dilution of 1 : 250 (4 *μ*g ml^−1^ final concentration), or anti-HPA1 monoclonal antibody (Insight Biopharmaceuticals, Rehovot, Israel) at a dilution of 1 : 60 (670 *μ*g ml^−1^ final concentration). An equivalent concentration of non-immune mouse serum was used as a negative control (DAKO, Kyoto, Japan). Sections were incubated with biotinylated rabbit anti-mouse antibody (1 : 400; 2 ng ml^−1^ final concentration; DAKO), and with peroxidase-labelled streptavidin (DAKO). Colour was developed using 3,3′-diaminobenzidine (Sigma-Aldrich, USA). Counterstaining was performed with Gill's haematoxylin. Samples were evaluated by microscopy and semiquantitatively analysed for heparanase expression (intense, moderate or absent staining).

## RESULTS

We have used two i*n situ* techniques to investigate the restricted distribution of biologically active HS to the tumour endothelium; the first question we addressed was whether there was evidence that HS synthesis occurred in tumours. We know that the molecular probe used to detect biologically active HS has an absolute requirement for the presence of 6-O-sulphate groups (Pye *et al*, 1998) and, therefore, we examined the distribution of the enzymes that catalyse the attachment of that moiety, the HS-6-O-sulphotransferases.

### *In situ* hybridisation locates HS6ST1 and HS6ST2 mRNA

**Figure 1:**
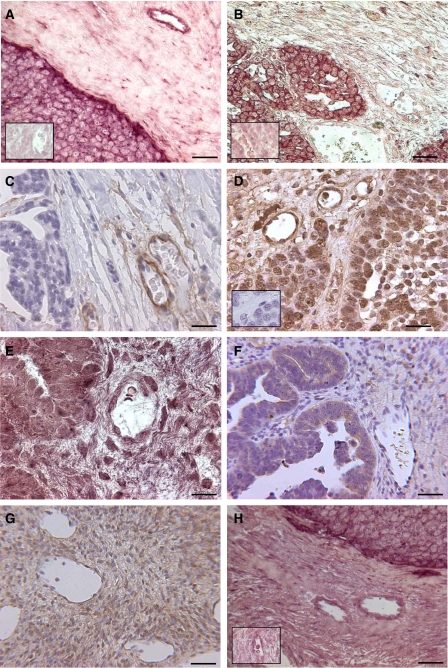
(**A**) HS6ST1 ISH shows RNA to be present in ovary tumour and endothelial cells, but absent in stroma and normal ovary (inset). (**B**) HS6ST2 ISH shows RNA to be present in ovary tumour cells, but absent in endothelial cells, stroma and normal ovary (inset). (**C**) 10E4, an anti-HS monoclonal antibody was used to demonstrate that intact HS chains were only present on endothelial cells in ovarian tumours. (**D**) 3G10 antibody was used to detect the HS stubs that remain after heparinase digestion. A negative control shows 3G10 staining to be negative without prior heparinase digestion (inset). (**E**) Heparanase ISH shows RNA to be present in ovary tumour cells, but absent in endothelial cells and stroma. (**F**) Heparanase IHC shows protein to be present in ovary tumour cells, but absent in endothelial cells and stroma. (**G**) Heparanase IHC on normal human ovary shows protein to be present at a low level in most cells. (**H**) HSULF-1 ISH shows RNA to be present in ovary tumour cells, but absent in endothelial cells, stroma and normal ovary (inset). Each scale bar represents 400 *μ*m.

Specific digoxigenin-labelled riboprobes for HS6ST1 and HS6ST2 mRNA were generated and used to investigate ten serous carcinomas and two normal ovaries. Our ability to study normal ovarian epithelium was compromised by the poor preservation of cellular architecture. Figure 1A shows HS6ST1 mRNA to be present in ovarian cancer and endothelial cells, but absent in stroma; all 10 tumours stained with similar intensities. Figure 1B shows HS6ST2 RNA to be present in ovarian cancer cells, but absent in endothelial cells and stroma and again all 10 tumours stained with similar intensities. Both HS6ST1 and HS6ST2 mRNAs were expressed at higher levels in tumour than in the normal ovaries examined (Figure 1A and B, inset).

We extended these studies by examining the distribution of the mRNA for 2-O-sulphotransferase and this closely mirrored that seen with the HS6ST2 mRNA, again suggesting that sulphated HS was made by ovarian cancer cells (data not shown).

### HS staining

In our previous study (Whitworth *et al*, 2005), which showed that biologically active HS was expressed by the endothelium (inferred by the binding of the FR1c-AP probe), the implication was that HS6ST1 would be confined to the endothelium. However, the above results contradict that hypothesis. Therefore, we carried out a series of IHC studies to determine whether HSPGs were expressed by the ovarian cancer cells.

The same 10 serous carcinomas and 2 normal ovaries were examined for the presence of HS using IHC. Figure 1C shows 10E4 staining, which indicated that intact sulphated HS chains were only present on endothelial cells in ovarian tumours. However Figure 1D shows 3G10 antibody staining, which detects the HS stubs that remain on the PG core protein after heparinase depolymerisation. The inset picture shows that 3G10 does not stain when the heparinase digestion step is omitted. With both 10E4 and 3G10, all 10 tumours stained with similar intensity. These experiments showed that carcinoma cells strongly bind to 3G10, suggesting that HSPGs were made by tumour cells, whereas the HS was subsequently degraded or modified to eliminate the 10E4 epitope.

### Investigation of heparanase (HPA1) mRNA and protein expression

We have shown that the HS6ST1 and 2 and HS2-OST biosynthetic enzyme RNA, essential for biologically active HS, were expressed by ovarian cancer cells although the HS 10E4 epitope, often used as a marker of HS, was not present on the cell surface. To investigate this further, we hypothesised that heparanase was present and may be responsible for the lack of 10E4 reactivity. A specific digoxigenin-labelled riboprobe for HPA1 was generated and used to investigate our panel of 10 serous carcinomas and two normal ovaries. Figure 1E shows heparanase RNA to be present in ovarian cancer cells, but absent in endothelial cells and stroma. Two of the 10 tumour specimens showed a lower expression of heparanase RNA. Because of the extensive processing of heparanase, we also used IHC and this localised HPA1 protein (Figure 1F) to tumour cells, but did not detect the enzyme in endothelial cells and only at a low level in stroma. All 10 tumours examined showed similar patterns of protein staining. In normal human ovary, immunohistochemistry showed that heparanase protein was present at a low level in most cells (Figure 1G). These experiments demonstrate that heparanase was strongly expressed by ovarian cancer cells, but was virtually absent from endothelial cells and normal ovarian tissues.

### *In situ* hybridisation locates HSULF-1 mRNA

HSULF-1 is an enzyme that removes 6-O-sulphate residues from cell-surface HS. Although the heparanase data might account for the distribution of biologically active HS, another possibility was that HSULF-1 might be responsible.

A specific digoxigenin-labelled riboprobe for HSULF-1 was generated and used to investigate ten serous carcinomas and two normal ovaries. Figure 1H shows HSULF-1 mRNA to be strongly expressed in ovarian tumour cells, but expressed at a much lower level in endothelial cells and stroma. The staining intensity was similar in all 10 tumours examined. HSULF-1 was expressed at higher levels in tumour than in the normal ovaries examined (Figure 1H, inset).

## DISCUSSION

In previous studies, we showed that stromal syndecan 1 was an adverse prognostic factor in ovarian cancer and that syndecan 3, which is usually found on neuronal tissue, was aberrantly expressed in the tumour vasculature (Davies *et al*, 2004). In a further investigation, we used a novel molecular probe, which demonstrated that endothelial HS was able to support receptor engagement when FGF2 was present; in other words, HS had the capacity to activate FGF2 (Whitworth *et al*, 2005). In this study we sought to explain the mechanism.

The findings from this study are summarised in Table 1. The HS epitope recognised by the antibody 10E4 was only detected on endothelial cells within ovarian tumour sections. Although the mRNAs of the synthetic enzymes were present in cancer cells, it was unclear whether HSPGs were expressed on the cell surface. By treating the tissues with heparinases to degrade cell-surface HS and then examining the distribution of bound, exogenously added 3G10 antibody, we were able to test whether the HSPG core proteins were present on the tumour-cell surface. These data showed widespread binding of the antibody in the tissue sections, suggesting that in cancer cells as well as in other cell populations present within the tumour HS-bearing PGs were expressed on the plasma membrane.

In a previous study (Whitworth *et al*, 2005), we showed that in ovarian tumours the distribution of biologically active HS revealed by the colocalisation of FGF2 and the FGF-receptor probe (FR1c-AP) was confined to the tumour endothelium. Whereas FGF2 binds to N and 2-O sulphated S-domains in HS, biologically active S-domains contain 6-O-sulphate moieties (Kan *et al*, 1993; Lindahl *et al*, 1998; Pye *et al*, 1998). Thus, the difference in binding requirements for the FR1c-AP probe and exogenous FGF2 is 6-O-sulphate on glucosamine; by comparing the distribution of the two probes we can examine the distribution of 6-O-sulphate. This is the moiety removed by the HSULF enzymes that we show here to be expressed strongly by tumour cells but weakly by the endothelial cells. Although our data apparently contradict those of Lai *et al* (2003), the differences have most likely arisen because in our study we determined the patterns of cellular expression of HSULF on tissue sections rather than PCR analysis of total tumour mRNA. However, our data also demonstrate that tumour cells and endothelial cells can be distinguished by their expression of HPA1; the high levels of this enzyme on tumour cells compared to its weak expression on the endothelium is likely to contribute to the differential reactivities of the HS found on the surfaces of these two cell populations. Thus, the combined effects of HSULF and HPA1 are probably responsible for the restricted distribution of biologically active cell-surface HS in ovarian cancer. These findings indicate that in ovarian tumours, FGF2 does not act directly on the malignant cells, rather it acts indirectly by promoting tumour angiogenesis.

Heparanase has been strongly correlated with metastatic potential in human cancer probably by disrupting the structure of basement membranes and by releasing HS saccharide growth factor complexes from the pericellular matrix enabling their unrestricted access to cell-surface receptors (Elkin *et al*, 2001). Our data again reinforce the importance of HS to the malignant phenotype and highlight the potential therapeutic advantage of targeting heparanase in human cancer.

## Figures and Tables

**Table 1:** Summary of results

	**Tumour**	**Stroma**	**Endothelium**
Endogenous FGF2^a^	Low	Low	Moderate
FGF2 binding^a^	Mild	Moderate	Moderate–strong
FR1c-AP^a^	None	Weak	Moderate–strong
HPA1	+++	+	−
HSULF-1	+++	+	+
HS6ST1	+++	+	+++
HS6ST2	+++	+	−
Intact HS (10E4)	−	−	+++
HS stubs (3G10)	+++	+	+++

a Findings of Whitworth *et al* (2005).
